# Genetic and physiological regulation of folate in pak choi (*Brassica rapa* subsp. *Chinensis*) germplasm

**DOI:** 10.1093/jxb/eraa218

**Published:** 2020-07-08

**Authors:** M J I Shohag, Yanyan Wei, Jie Zhang, Ying Feng, Michael Rychlik, Zhenli He, Xiaoe Yang

**Affiliations:** 1 Ministry of Education (MOE) Key Laboratory of Environmental Remediation and Ecosystem Health, College of Environmental and Resources Science, Zhejiang University, Hangzhou, China; 2 Department of Agriculture, Bangabandhu Sheikh Mujibur Rahman Science and Technology University, Gopalganj, Bangladesh; 3 Indian River Research and Education Center, Institute of Food and Agricultural Sciences, University of Florida, Fort Pierce, FL, USA; 4 Cultivation Base of Guangxi Key Laboratory for Agro-Environment and Agro-Products Safety, College of Agriculture, Guangxi University, Nanning, China; 5 International Research Center for Environmental Membrane Biology, Department of Horticulture, Foshan University, Guangdong, China; 6 Chair of Analytical Food Chemistry, Technische Universitat Munchen, Lise-Meitner-Str. 34, Freising, Germany; 7 University of Essex, UK

**Keywords:** *Brassica rapa* L, breeding, folate, folate metabolism genes, natural variation, nutrient composition, physiology

## Abstract

Folates are one of the essential micronutrients for all living organisms. Due to inadequate dietary intake, folate deficiency remains prevalent in humans. Genetically diverse germplasms can potentially be used as parents in breeding programs and also for understanding the folate regulatory network. Therefore, we investigated the natural genetic diversity of folates and their physiological regulation in pak choi (*Brassica rapa* subsp. *Chinensis*) germplasm. The total folate concentration ranged from 52.7 μg 100 gFW^–1^ to 166.9 μg 100 gFW^–1^, with 3.2-fold variation. The main folate vitamer was represented by 5-CH_3_-H_4_folate, with 4.5-fold variation. The activities of GTP cyclohydrolase I and aminodeoxy chorismate synthase, the first step of folate synthesis, were high in high folate accessions and low in low folate accessions. Analysis of the transcription levels of 11 genes associated with folate metabolism demonstrated that the difference in folate concentrations may be primarily controlled at the post-transcriptional level. A general correlation between total folate and their precursors was observed. Folate diversity and chlorophyll content were tightly regulated through the methyl cycle. The diverse genetic variation in pak choi germplasm indicated the great genetic potential to integrate breeding programs for folate biofortification and unravel the physiological basis of folate homeostasis in *planta*.

## Teaser

The diverse genetic variation in pak choi germplasm provides the fundamental basis for breeding folate-rich pak choi varieties and helps to unravel the physiological regulation of folate homeostasis *in planta*.

## Introduction

Folate (vitamin B_9_) is one of the most nutritionally significant vitamins for human, as it is involved in one-carbon (C1) metabolism reaction by providing C1 units for biosynthetic and regulatory functions. One-carbon units are essential for universal methylation reactions on DNA, proteins, and lipids, plus biosynthesis of nucleic acids and amino acids (Suh *et al.*, 2001). Therefore, folate deficiency in diets manifests with some serious chronic disorders, including neural tube defects (NTDs) such as spina bifida and anencephaly in infants, Alzheimer’s disease, Down syndrome, megaloblastic anemia, occlusive vascular disease, and colon cancer, in adults (Lucock, 2000; Lucock and Yates, 2005; Blom *et al.*, 2006). Synthetic folic acid fortification in staple food has created a rising concern because of its probable antagonism against anticancer medicine (Dary, 2009), promotion of existing tumors (Kim, 2004), masking of B_12_ deficiency (Reynolds, 2006), and exposure of unmetabolized folic acid in the blood circulation (Wright *et al.*, 2007). In contrast to synthetic folic acid supplementation and fortification, increasing natural folate content in food crops through biofortification has therefore become a high priority research area.

**Fig. 1.**
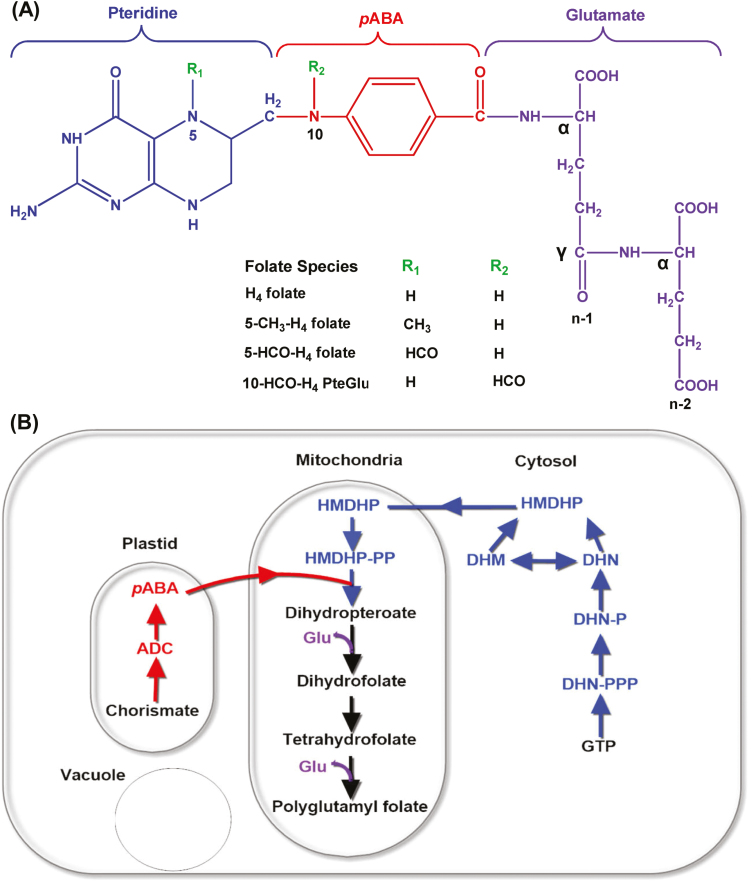
The structure and biosynthetic pathway of folates in plants. (A) Chemical structure of tetrahydrofolate (H_4_folate). Folates are tripartite molecules consisting of pteridine (blue), *p*ABA (red), and glutamate moieties (violet). One-carbon units at various levels of oxidation (methyl, methylene, formyl) are attached to the N_5_ and/or N_10_ position. Plant folates have γ-linked polyglutamyl tails of up to approximately six residues attached to the γ-carboxyl group of the first glutamate residue. (B) The plant folate biosynthesis pathway. The pteridine pathway leading to hydroxymethyldihydropterin (HMDHP) is shown in blue, the pathway leading to *p*-aminobenzoate is shown in red, and the remaining steps localized in the mitochondria are in black. ADC, aminodeoxychorismate; DHN, dihydroneopterin; -P, monophosphate; -PP, pyrophosphate; -PPP, triphosphate; DHM, dihydromonapterin.

The folate molecule is formed with three building blocks, a pteridine ring, a *p*-amino benzoic acid (*p*ABA), and a glutamate chain (Fig. 1A). C1 units such as methyl (–CH_3_), formyl (–CHO), and methylene (–CH_2_–) can be joined either at the N_5_ position of the pteridine ring or at the N_10_ position of the *p*ABA molecule, or bridged between these two positions (Hanson and Gregory, 2011). I*n planta*, H_4_folate is synthesized in the mitochondria, through a tri-compartmentalized biosynthetic pathway. Pteridine molecules are synthesized from GTP in the cytosolic compartment through sequential enzymatic reactions of GTP cyclohydrolase I (GCHI) (Basset *et al.*, 2002), dihydroneopterin triphosphate (DHNTP) diphosphatase (DHNTP-PPase), and DHN aldolase (DHNA) (Goyer *et al.*, 2004). DHNA also catalyzes epimerization of DHN to dihydromonapterin (DHM), which is also split into hydroxylmethyldihydropterin (HMDHP). *p*ABA is synthesized in the chloroplasts from chorismate through sequential enzymatic reactions of aminodeoxychorismate (ADC) synthase (ADCS) and ADC lyase (ADCL), and then linked together with cytosolic pteridine in the mitochondria to produce H_2_pteroate. In mitochondria, HMDHP is first pyrophosphorylated by HMDHP pyrophosphokinase (HPPK) and then condensed with *p*-ABA by dihydropteroate synthase (DHPS). A glutamate chain is connected to H_2_pteroate to produce H_2_folate and subsequent production of H_4_folate by the activity of dihydrofolate synthetase (DHFS) and dihydrofolate reductase (DHFR), respectively (Bekaert *et al.*, 2008). The polyglutamyl tail is then added by folylpolyglutamate synthases (FPGS) to form polyglutamyl folate (Fig. 1B), and the tail can be removed by γ-glutamyl hydrolase (GGH). The resulting folate molecules participate in various forms of C1 metabolism. It has been proposed that GGHs, *p*-ABA-glucose hydrolase, and pterin aldehyde reductase are involved in a salvage pathway for folate degradation (Hanson and Gregory, 2011). *In planta*, folate molecules play a vital role in photorespiration, nitrogen metabolism, and the biosynthesis of chlorophyll, lignin, betaine, and alkaloids (Hanson and Roje, 2001; Jiang *et al.*, 2013).

Folate biofortification through either breeding or metabolic engineering is the main strategy to fight against folate deficiency and is potentially a safe, cost-effective, and more sustainable strategy than the so-called supplementation strategy (Rebeille *et al.*, 2006). Therefore, folate biofortification in food crops through plant breeding or metabolic engineering has become the sustainable alternative to alleviate folate deficiency worldwide. In particular, fundamental research efforts have been made to boost folate concentration in food crops by metabolic engineering (DellaPenna, 2007; Blancquaert *et al.*, 2014). To date, metabolic engineering approaches have focused on increasing the folate metabolic flux (Díaz de la Garza et al., 2004, 2007; Storozhenko *et al.*, 2007; Nunes *et al.*, 2009; Blancquaert *et al.*, 2013) and complexing folate to folate-binding proteins to improve folate stability (Blancquaert *et al.*, 2015) through the biosynthetic pathway. Despite good success of these strategies, each of these has its own drawbacks and limitations. In the folate biosynthesis pathway, all the enzymes and their compartmentation are fairly well known, but regulation of the pathway is not yet well understood, which obstructs the engineering strategy (Blancquaert *et al.*, 2013). Exploiting natural genetic variation of folate contents within the crop species by integrating genomics with the conventional breeding approach is not only a paradigm for folate improvement of crops, but also useful to understand the molecular and physiological basis of folate homeostasis in plant species (Rebeille *et al.*, 2006; Bekaert *et al.*, 2008).

Plants are the main sources of folate in human nutrition. Leafy green vegetables, certain fruits, and legumes are the abundant sources of dietary folates (Scott *et al.*, 2000), whereas folate contents are very low in cereal crops (USDA, 2019). Because vegetables are the main contributors to natural folate intake worldwide, increasing vegetable intake may be the best way to lower folate deficiency without health concerns. Pak choi (*Brassica rapa* subsp. *Chinensis*) is a nutritionally and economically important leafy green vegetable gaining acceptance all over the world (AVRDC, 2000; Yu *et al.*, 2010). This morphologically highly diverse *B. rapa* genus (Yu *et al.*, 2010) is an excellent source of carotenoids, especially lutein and zeaxanthin, β-carotene, calcium, iron, and glucosinolates (Hanson *et al.*, 2009), and it has been recently identified as having a high folate content, close to that of spinach (Shohag, *et al.*, 2012b).

The aim of this study was first to investigate the natural genetic variation in folate levels and their vitamers in a comprehensive number of pak choi germplasm/accessions grown under controlled environmental conditions. Secondly, since little is known about the physiological regulation of the folate biosynthesis pathway in plants, we analyzed the enzyme activity, expression of folate metabolism genes in naturally diverse high and low folate content germplasms, and the resultant accumulation of folate biosynthetic precursor and C1 metabolites to elucidate the naturally controlled folate homeostasis network.

## Materials and methods

### Plant materials and culture

#### Collection of pak choi accessions

Seventy-four accessions of pak choi (*B. rapa* subsp. *Chinensis*) were selected according to geographical origin, seasonal group, and horticultural group. The collection included 11 landraces (all from China) and 69 accessions of commercially available varieties (7 from Japan, 62 from China). Details of the accession name, acronyms, and origin are shown in Supplementary Table S1 at *JXB* online. Seed stocks were obtained from either the Beijing Vegetable Research Center (China) or local seed markets.

#### Seed germination

Seeds were surface sterilized by 0.07% sodium hypochlorite (NaOCl) for 30 min and rinsed in deionized water four times. They were then placed in aerated deionized water (23±3 °C) and soaked overnight. The soaked seeds of each accession were germinated on moistened quartz in black plastic trays for 5 d. Uniform seedlings were then transplanted in black plastic pots (18×18×18 cm) filled with growth medium (Metro-Mix 360, Sun Gro Horticulture, Canada).

#### Culture conditions

Pak choi accessions were grown in controlled-environmental conditions as described previously (Shohag *et al.*, 2011) with slight modification. After transplanting, 10 healthy plants of each accession were permitted to grow per pot; the remainder were thinned. Pots were randomly placed in a growth chamber (http://www.zjuee.cn) and their position was altered every third day. Plants were maintained on a 12 h photoperiod of 200 µmol m^−2^ s^−1^ photosynthetically active radiation (incandescent and fluorescent lamps) with a 22±0.5 °C/18±0.5 °C day/night temperature regime. Relative humidity was maintained at 60±5%. Plants were initially irrigated with deionized water, and subirrigated daily with nutrient solution after transplanting, as described previously (Shohag *et al.*, 2011). Before application, the pH of the nutrient solution was adjusted to 5.7 with 0.1 N NaOH or 0.1 N HCl.

#### Sampling procedure

Plants were harvested at 6 weeks of age, when they reached commercial maturity. Plants were excised 0.5 cm above the base. Aboveground parts of harvested material including both mature and immature leaves (leaf blades and petioles) were weighed to determine the fresh weight. At least six randomly selected plants of each accession were cut into small pieces using sharp scissors and combined together to make a composite sample representing the accession for determination of fresh weight, dry weight, folate, enzyme, gene expression, precursor, and C1 metabolite analysis.

### Folate measurement

#### Folate extraction

Folates were extracted according to our previously described report with some modifications (Shohag *et al.*, 2012b). In brief, 1 g (FW) of frozen composite samples was ground in liquid nitrogen, immediately transferred into a 10 ml screw-capped tube (Sangon Biotech Co., Ltd., Shanghai, China), and then 4 ml of extraction buffer [50 mM phosphate buffer containing 1.0% l(+)-ascorbic acid (w/v) and 0.1% 2,3-dimercapto-1-propanol (BAL) (v/v) at pH 6.7, with freshly prepared internal standard (MTX concentration 0.03 µg ml^–1^)] was added. The samples were flushed with argon gas and homogenized with a ball-mill (Scientz-48, Ningbo Scientz Biotechnology Co., Ltd, Ningbo, China) followed by boiling in a water bath at 100 °C for 12 min, rapid cooling on ice, and ultracentrifugation at 27 000 *g* for 20 min at 4 °C. For deconjugation of polyglutamylated folates, 75 μl of rat serum and 40 μl of chicken pancreas solution were added to 500 μl of the extraction solution, flushed with argon gas before capping, and then incubated on a rotary water bath at 37 °C for 2 h as described in our previous report (Shohag *et al.*, 2012a). An additional treatment of 5 min at 100 °C was carried out to inactivate the enzyme, again followed by cooling on ice. The samples were then centrifuged again at 18 000 *g* for 15 min. For ultrafiltration, 0.5 ml of supernatant solution was added to the upper chamber of the 5 kDa molecular weight filter cut-off membrane filter (Millipore, Carrigtwohill, Co., Cork, Ireland) and centrifuged at 12 000 *g* for 12 min at 4 °C.

#### Folate analysis

Folates were analyzed by the Waters ACQUITY UPLC system (Waters Corporation, Milford, MA, USA) coupled with a tandem quadrupole mass spectrometer (Waters Xevo TQ-S, Waters Corporation) as described in our recently developed method (Shohag *et al.*, 2017). MS was operated in the positive electrospray ionization (ESI) mode and the source parameters of the mass spectrometer were optimized automatically with flow injection analysis. The column oven temperature was maintained at 40 °C, and the autosampler was maintained at 4 °C. The separation of folates was performed on an ACQUITY UPLC BEH, C_18_ column, dimension 2.1×50 mm, 1.7 µm particle size (Waters Corporation) connected with a VanGuard pre-column C18, dimension 5×2.1 mm; 1.8 μm particle size (Waters Corporation). The flow rate was 0.4 ml min^–1^; the injection volume was 2 μl with a total running time of 5 min. The mobile phase was a binary gradient (Supplementary Table S2) mixture of eluent A (0.1% formic acid in water) and eluent B (0.1% formic acid in acetonitrile). For these analyses, two to four multiple reaction monitoring (MRM) transitions were used for each vitamer (Supplementary Table S3). Detailed MS/MS conditions are shown in Supplementary Table S4. Certified reference material (Mixed vegetables, BCR-485) was analyzed in each batch of samples to check the accuracy and for quality control. The sum of folates, expressed as folic acid, was 289.3±14.22 µg 100 g^–1^ (*n*=5) in the range of the certified value 315±28 µg 100 g^–1^. Sensitivity was assessed by evaluating the LOD (S/N>3) and LOQ (S/N>10) values. Intra- and interday precision in BCR-485 were <7.2% relative standard deviation (RSD), and were obtained on three different days for all folate forms. Recovery and matrix effect were assessed similarly as described in our previous report (Shohag *et al.*, 2017).

### Enzyme activity assay

#### GTP cyclohydrolase I (GCHI) assay

The GCHI enzyme assay was adapted from Hossain *et al.* (2004) and McIntosh *et al.* (2008). The enzyme assay contained 5 mg of protein, 0.5 mM Tris–HCl (pH 7.5), 1 mM DTT, 50 mg BSA, and 1 mM GTP in a total volume of 50 ml, and was incubated for 1 h at 37 °C. The product of the reaction, dihydroneopterin triphosphate, was subsequently oxidized and dephosphorylated to neopterin prior to LC-MS analysis. Dephosphorylation using calf intestinal phosphatase was performed in a pH range of 7.5–8.0 to avoid production of a phosphatase-resistant cyclic dihydroneopterin monophosphate. LC-MS analysis was performed on an Agilent 1200 Series coupled with a 1100 Series Mass Selective Detector. Reaction products including neopterin were separated on a Phenomonex 5 µm C18 column (150×4.6 mm) and eluted isocratically using 0.5% acetonitrile with a flow rate of 1 ml min^–1^, a column temperature of 40 °C, and a 10 µl injection volume. Detection of neopterin was confirmed by monitoring absorption maxima at 280 nm and 340 nm. Neopterin was identified by MS in scanning mode in a mass range of 100–600 *m/z*. Neopterin was used as the reaction standard and was assayed under the same experimental conditions.

#### Aminodeoxy chorismate synthase (ADCS) assay

Assays of ADCS activity were modifications of a published procedure (Nichols *et al.*, 1989). Briefly, standard assays (100 µl) contained 50 mM Tris–HCl (pH 7.5), 10 mM MgCl_2_, 10 mM DTT, 5 mM l-glutamine, 100 µM chorismate (glutamine-dependent assays), or 40 mM triethanolamine (pH 8.5), 26 mM (NH_4_)_2_SO_4_, 8 mM MgCl_2_, 4 mM DTT, and 80 µM chorismate, and were run at 37 °C for 30–120 min. Desalted extract (7 µg of protein) was added when indicated. Reactions were stopped with 20 µl of 75% (v/v) acetic acid, incubated on ice for 1 h, and the solution was centrifuged (15 000 *g*, 4 °C, 20 min). Supernatants (50 µl) were injected onto a Supelco Discovery C_18_ column (5 m, 250×4.6 mm) and eluted isocratically with 0.5% acetic acid containing 20% (v/v) methanol at a flow rate of 1 ml min^–1^. The peak was detected by fluorescence (290 nm excitation, 340 nm emission) and quantified relative to standards.

### Gene expression analysis

#### Total RNA isolation and cDNA synthesis

The same plant material was used for total RNA isolation as for folate analysis. Total RNA was extracted from frozen composite sample using RNAiso Plus (Takara Bio Inc., Shiga, Japan) according to the manufacturer’s protocol. To eliminate genomic DNA, extracted RNA was treated with the gDNA Eraser supplement provided with the PrimeScript RTreagent Kit (Takara Bio Inc.). UV absorption spectrophotometry and gel electrophoresis were performed to test RNA quality and purity as described in the product manual. cDNA was synthesized from 5 μg of total RNA using the PrimeScript RTreagent Kit (Takara Bio Inc.) following the manufacturer’s procedures. To confirm the absence of genomic DNA, all cDNA samples were tested with specific primers of the gene encoding pak choi *actin.*

#### Quantitative real-time PCR (qRT-PCR) analysis

The transcription levels of *GCHI*, *DPP*, *DHNA*, *HPPK/DHPS*, *ADCS*, *ADCL*, *DHFS*, *DHFR*, *FPGS*, *GGH1*, and *GGH2* were analyzed by using qRT-PCR with the ABI StepOne PCR System (Applied Biosystems, Foster City, CA, USA). All reactions were performed using the Fast Start Universal SYBR Green Master Mix (Applied Biosystems) following the manufacturer’s recommended procedures. The gene-specific primers used for qRT-PCR are listed in Supplementary Table S5. The lowest folate content accession was chosen as a calibrator for each group of samples and assigned a nominal value of 1.0. PCRs were performed using ~1 μl of cDNA (50 ng μl^–1^ of total RNA), 0.3 μM of each specific primer, and 10 μl of 1× SYBR Green PCR master mix (Applied Biosystems) in a 20 μl volume. A negative control was included using water as a template in each reaction. The reaction mixtures were initially denatured at 95 °C for 10 min, followed by a quantification program of 40 cycles of 95 °C for 15 s and 60 °C for 60 s. At the end of each run, melting curve analyses were conducted following the instrument instructions by slowly increasing the temperature from 60 °C to 95 °C to ensure the specificity of the primer and the purity of the amplified product. Relative expression levels were calculated using the ΔΔCt method (Pfaffl, 2001) and normalized Ct data obtained from a target gene with Ct values from the *actin* gene as an internal control. The Ct values presented are the means of three independent biological replicates, and each reaction had three technical replicates.

### Folate precursor analysis

The folate precursors pteridine and *p*ABA were analyzed as described previously (Díaz de la Garza *et al.*, 2007) with several modification. In brief, 1 g (FW) of frozen composite samples was ground in liquid nitrogen, immediately transferred into a 10 ml graduated screw-capped microtube (Sangon Biotech Co., Ltd), and then 8 ml of cold methanol (stored at −20 °C) was added to each tube and homogenized on a bench-top ball-mill (Scientz-48, Ningbo Scientz Biotechnology Co., Ltd, Ningbo, China). After centrifugation for 15 min at 1800 *g*, the supernatant was transferred to a 15 ml tube, and another 2 ml of methanol was added to the residue for a second extraction. Subsequently, both supernatants were combined.

#### Pteridine analysis

A 600 μl aliquot of the supernatant was combined with 250 μl of CHCl_3_ and 50 μl of water and shaken in a rotary shaker for 40 min before another 225 μl of CHCl_3_ and 340 μl of water were added. After shaking for 20 min and centrifuging to break the emulsion, the aqueous phase was removed, dried under nitrogen flow, and re-dissolved in 200 μl of water. Samples were oxidized by 1% I_2_ and 2% KI (w/v) in 1 M HCl and incubated in darkness for 1 h; excess I_2_ was then removed by adding 10 μl of 5% (w/v) Na-ascorbate. The oxidized samples were separated by an LC system (Agilent 1200 Series, Agilent Technologies, Germany) with a Zorbax SB, C_18_, column 250×4.6 mm, 5 μm (Agilent Technologies, Santa Clara, CA, USA) eluted isocratically with 10 mM Na-phosphate (pH 6.0) at a flow rate of 1.5 ml min^–1^. Peaks were detected by a fluorescence detector (350 nm excitation and 450 nm emission) and identified by reference to standards and by spectral properties. Pteridine contents were expressed as hydroxymethylpterin (HMPT) equivalents after calibration with authentic HMPT (fluorescence responses of the various pteridines differ by <38%). HMPT-pyrophosphate was estimated from the difference in HMPT values before and after treatment of the pteridine extract with alkaline phosphatase.

#### *p*ABA analysis

A 5 ml aliquot of the supernatant was used for analysis of total *p*ABA (free *p*ABA and *p*ABA glucose ester) by acid hydrolysis. The methanolic layer was dried under a nitrogen gas flow at 35 °C and then 2 ml water was added followed by ultrasonication for 5 min. For acid hydrolysis, 0.1 ml of 2 N HCl was added to glass tubes each containing 0.8 ml sample solution. After capping, the tubes were incubated at 80 °C for 2 h. After cooling down the solution, 0.1 ml of 2 N NaOH was added for neutralization. Finally, the sample solutions were subjected to ultrafiltration at 12 000 *g* for 30 min on a 5 kDa molecular weight cut-off membrane filter (Millipore) before LC analysis. Separation of *p*ABA was achieved with a Zorbax SB, C_18_, column 250×4.6 mm, 5 μm particle size (Agilent Technologies), at 42 °C. For the detection and quantification of *p*ABA, a fluorescence detector (excitation/emission 290/340 nm) and a UV detector (290 nm) were used. The mobile phase consisted of eluent A (0.1% formic acid in water) and eluent B (0.1% formic acid in methanol). The starting eluent was 98% A mixed with 2% B; the proportion of B was increased linearly to 17% in 15 min, then to 95% in 15 min. The mobile phase was immediately adjusted to its initial composition and held for 10 min to re-equilibrate the column. The flow rate of the mobile phase was 0.7 ml min^–1^, and the injection volume was 50 μl. Under these conditions, the retention time of *p*ABA was 12.8 min. A *p*ABA stock solution containing 1 mg ml^–1^*p*ABA in water was serially diluted in water to prepare the standard solutions ranging from 0.01 µg ml^–1^ to 10 µg ml^–1^.

### Measurement of C_1_ metabolites

#### Homocysteine, *S*-adenosylmethionine, and *S*-adenosylhomocysteine

Homocysteine (Hcy), *S*-adenosylmethionine (AdoMet), and *S*-adenosylhomocysteine (AdoHcy) were extracted and derivatized as described in a previous report (Van Wilder *et al.*, 2009) and analyzed using a Waters ACQUITY UPLC system (Waters Corporation) coupled with a tandem quadrupole mass spectrometer (Waters Xevo TQ-S, Waters Corporation) as described by Wang *et al.* (2014) with minor modifications. The separation of Hcy, AdoMet, and AdoHcy was performed on an ACQUITY UPLC BEH, C_18_ column, dimension 2.1×50 mm, 1.7 µm particle size (Waters Corporation) connected with a VanGuard pre-column C18, dimension 5×2.1 mm; 1.8 μm particle size (Waters Corporation). The column oven temperature was maintained at 40 °C, and the flow rate was 0.4 ml min^–1^; the injection volume was 5 μl with a total running time of 7 min. The mobile phase was a binary gradient (Supplementary Table S6) mixture of eluent A (0.1% formic acid in water) and eluent B (0.1% formic acid in acetonitrile).

#### Analysis of soluble amino acids

Soluble amino acids were determined following our previously developed protocol (Wei *et al.*, 2012). The amino acids were quantified using an automatic amino acid analyzer (L-8900, Hitachi, Tokyo, Japan). The analysis was performed according to the manufacturer’s standard protocols.

### Chlorophyll determination

Fresh composite sample (100 mg) was sliced into small pieces and extracted in the presence of 80% (v/v) acetone for 24 h. The absorbance of the extraction solution at 647 nm and 664 nm was recorded using a UV-vis spectrophotometer (Lambda 35, UV-vis spectrophotometer, PerkinElmer Ltd). Chlorophyll concentration was calculated using the equation described by (Lichtenthaler, 1987).

### Nutrient analysis

#### Determination of mineral concentrations

The composite samples were dried at 65 °C and then thoroughly homogenized by a bench-top ball-mill (Retsch, MM-301, Germany). The homogenized samples (0.3 g) were placed in a digestion tube and digested with nitric acid (2 ml) and hydrogen peroxide (0.5 ml). After cooling, the digestion solution was transferred to a 25 ml volumetric flask, with the volume made up with deionized water. The concentrations of zinc (Zn), iron (Fe), calcium (Ca), and selenium (Se) in the sample were determined by inductively coupled plasma MS (ICP-MS, Agilent 7500a, Agilent Technologies, CA, USA) following our previously described method (Wei *et al.*, 2012). In the experiment, the standard reference material, cabbage leaf (SRM 1568a), from the National Institute of Standards and Technology (Gaithersburg, MD, USA) was used to check the accuracy of Zn, Fe, and Ca analysis. The measured values were 19.7±0.2 mg kg^–1^ for Zn, 6.9±0.3 mg kg^–1^ for Fe, and 114.5±1.2 mg kg^–1^ for Ca, which were in accordance with the certified ranges of 19.4±0.5 mg kg^–1^ for Zn, 7.4±0.9 mg kg^–1^ for Fe, and 118±6 mg kg^–1^ for Ca.

#### Protein content determination

Dry composite samples were analyzed for protein content by determination of total nitrogen. Pak choi samples (0.5 g) were digested by 20 ml of H_2_SO_4_, distilled in KjelFlex K-360 (Buchi, Flawil, Switzerland) with 40% (w/v) NaOH and 2% (w/v) boric acid (methyl red and bromcresol green used as an indicator solution), and then titrated with 0.02 mM H_2_SO_4_. The protein content was subsequently calculated by multiplying nitrogen content by a conversion factor of 5.95 (Wei *et al.*, 2012).

#### Vitamin C analysis

Vitamin C contents, ascorbic acid, and dehydroascorbic were determined by LC after reduction of dehydroascorbic to ascorbic acid as described in our previous report (Shohag *et al.*, 2012a). In brief, 1 g (FW) of frozen composite samples was ground in liquid nitrogen, immediately transferred into a 10 ml graduated screw-capped micro-tube (Sangon Biotech Co., Ltd), and then 5 ml of 4.5% ice-cold metaphosphoric acid (MPA) was added. The content of the tube was then homogenized on a bench-top ball-mill (Scientz-48, Ningbo Scientz Biotechnology Co., Ltd) and centrifuged at 10 000 *g* for 10 min. The extraction process was repeated twice, and the volume was made up to 20 ml by MPA. For reduction of dehydroascorbic, an aliquot of the extract was added with 1% DTT solution and allowed to react for 30 min. Samples were analyzed using an LC system (Agilent 1200 series, Agilent Technologies, Germany) following an isocratic method with a flow rate of 1.0 ml min^–1^. For separation of ascorbic acid, a Zorbax SB, C_18_ column, 250×4.6 mm with a particle size of 5 μm (Agilent Technologies), was used. Absorbance was measured at 254 nm using a UV detector. The mobile phase composition and calibration method were similar to those described in our previous report (Shohag *et al.*, 2012a).

### Statistical analysis

The obtained data were subjected to ANOVA. The data are expressed as mean ±SD, and the differences between the germplasms were compared by using a multiple-range test (least significance difference) at a *P*<0.05 probability level (Duncan’s test). The relationships between the total folate content, biomass yield, or metabolites were analyzed using Pearson correlation analysis. All the statistical analysis was performed using the R package (https://www.r-project.org/).

## Results

### Total folate and vitamer distribution in diverse pak choi accessions

**Fig. 2.**
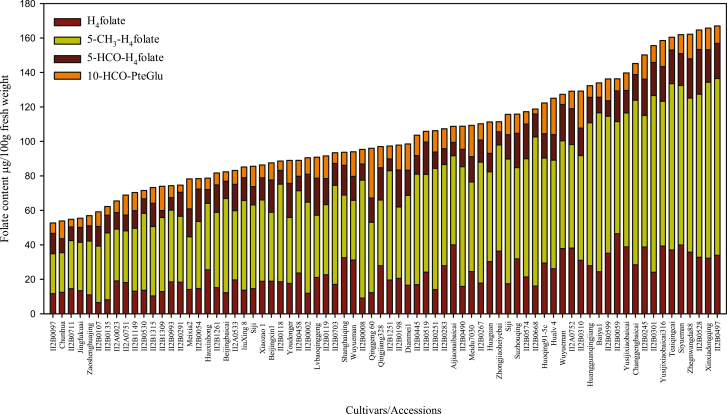
Folate composition measured in 74 pak choi (*Brassica rapa* subsp. *Chinensis*) accessions/cultivars grown under controlled conditions. Folate was extracted from at least six plants to form a composite sample, and duplicate determinations were made on each extract. Data are means ±SD of all determinations (Supplementary Table S1).

The pak choi accessions selected for this study displayed substantial variation for nearly all morphological characteristics (Supplementary Table S1) and thus were suitable materials for investigating the genetic and physiological regulation of folate content. The folate content among the germplasms ranged from 52.7 μg 100 gFW^–1^ to 166.9 μg 100 gFW^–1^ (Fig. 2; Supplementary Table S1), which was a 3.2-fold variation from the lowest to highest folate level, with a mean value of 103.4 μg 100 gFW^–1^.

**Fig. 3.**
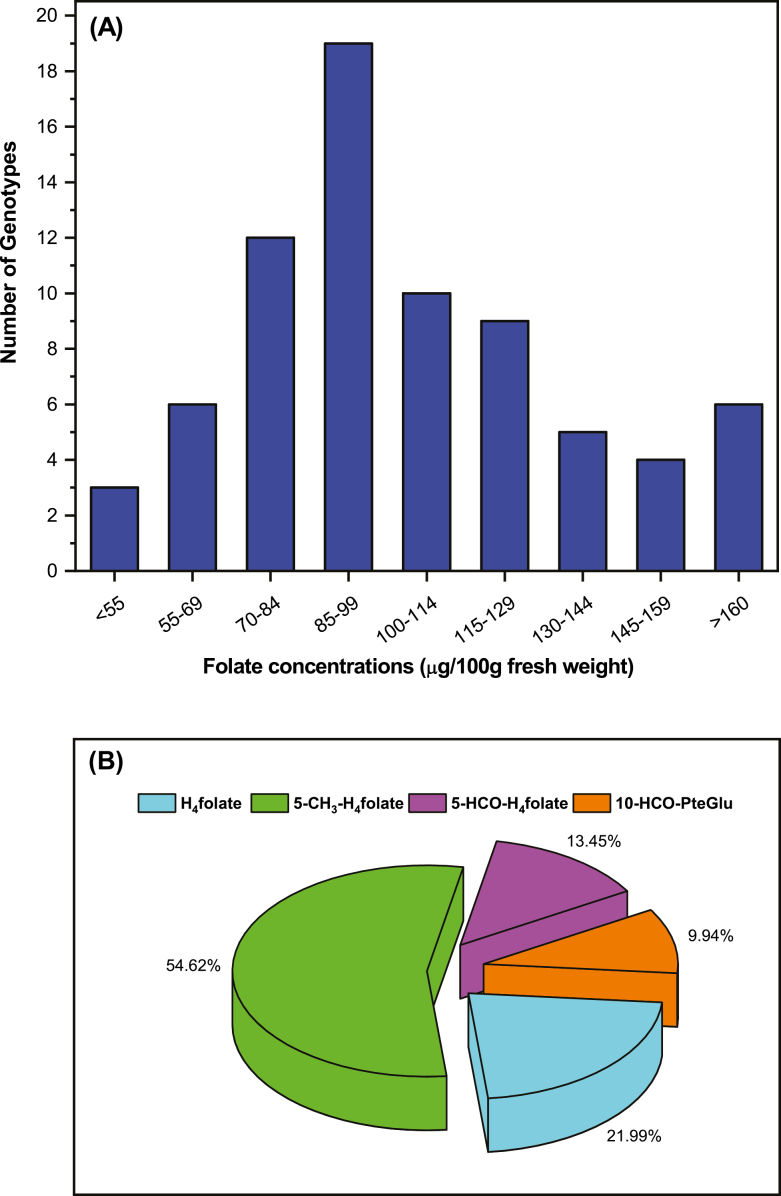
Frequency and folate vitamer distributions. (A) Frequency of pak choi (*Brassica rapa* subsp. *Chinensis*) cultivars/accessions in relation to total folate content (Supplementary Table S7). (B) Folate vitamer distribution in *Brassica rapa* cultivars/accessions.

When the range in the total folate content was divided into intervals of 14 μg 100 gFW^–1^, giving nine groups, the results demonstrated that each of the groups include 3–19 accessions (Fig. 3A; Supplementary Table S7). This comparison thus demonstrated that most of the accessions were distributed near their mean folate content. However, the largest number of accessions (19 accessions) fell within the interval of 85–99 μg 100 gFW^–1^, which means that 25% of accessions had folate contents within this range. The second largest group (12 accessions) fell within the interval of 70–84 μg 100 gFW^–1^, which means that 16% of accessions had folate contents in this range. The third largest group (10 accessions) fell within the interval of 100–114 μg 100 gFW^–1^, and covers 13% of total accessions screened. These three groups covered 54% of the accessions, which were the medium sources of folate content, with the range of 70–114 μg 100 g FW^–1^. Above and below the range of 70–114 μg 100 g FW^–1^, nine and six accessions fall into 115–129 μg 100g FW^–1^ and 55–69 μg 100 gFW^–1^, and accounted for 12% and 8% of the total accessions screened, respectively. A total of five accessions, falling into the range of 130–144 μg 100 gFW^–1^, were the moderate sources of folate and accounted for 6% of the total accessions analyzed. A total of four accessions accounted for 5% of total accessions, falling into the range of 145–159 μg 100 gFW^–1^, and were the moderate high folate content accessions. Furthermore, three accessions fell below 55 μg 100 gFW^–1^. This group was the poorest folate-containing accession, accounting for 4% of total accessions evaluated. Finally, six accessions exceeded 160 μg 100 gFW^–1^. These were the highest folate content accessions, accounting for 8% of the total accessions evaluated.

The highest folate content of 166.9±2.1 μg 100 gFW^–1^ was observed in accession number II2B0497, which originated from Ningxia. Other accessions in the topmost group were from Jiangxi (Xinxiadongqing), Hubei (II2B0528), and Shangdong (Zhegnwangda88). On the other hand, the lowest folate content of 52.7±0.5 μg 100 gFW^–1^ was observed in accession number II2B0097, which originated from Zhangsu. Accessions in the bottom group, however, originated from Beijing, Henan, Jiangsu, and Japan. Overall, a comparison of total folate contents of the accessions with the information on their origin thus showed that the highest and lowest folate sources were evenly distributed among their geographical origins.

Folate vitamer distribution was analyzed (Fig. 3B), because each of the vitamers differs in its bioavailability and stability. 5-CH_3_-H_4_folate was the amplest vitamer observed in pak choi and varied from 23 μg 100 gFW^–1^ to 102.5 μg 100 gFW^–1^, contributing on average 54.6% of all of the analyzed vitamers, varying from 44% to 61% depending upon the accessions evaluated. On the other hand, H_4_folate, 5-HCO-H_4_folate, and 10-HCO-folic acid were contributing 21.9, 13.4, and 9.9% of the analyzed vitamer, respectively, which were 45% of the total vitamers. The more stable vitamers 5-HCO-H_4_folate and 5-CH_3_-H_4_folate contributed 68% of the total folate pool in pak choi.

### Association of the total folate content with biomass yield

**Fig. 4.**
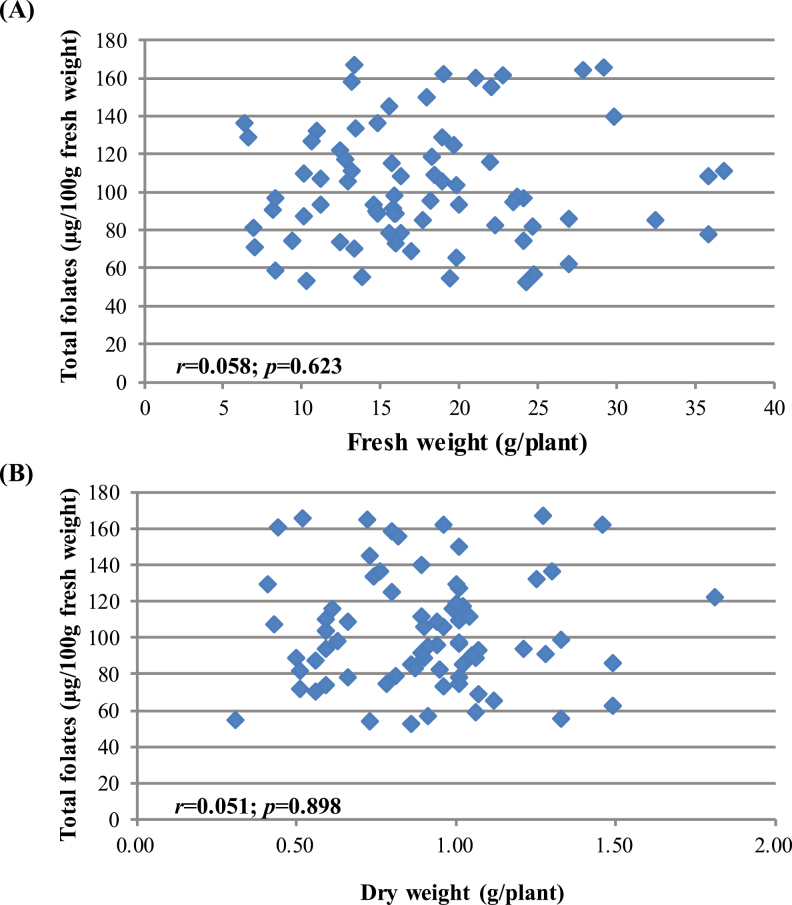
Correlation of total folate contents and shoot biomass yield in pak choi (*Brassica rapa* subsp. *Chinensis*) cultivars/accessions. (A) Fresh weight basis (not significant). (B) Dry weight basis (not significant).

A substantial number of pak choi accessions were compared in this highly controlled experiment. Interactions of total folate content and biomass yield were also examined on both a fresh weight (Fig. 4A) and a dry weight (Fig. 4B) basis. There were no or little correlations (*r*=0.058; *r*=0.015) between these two parameters and were statistically insignificant (*P*=0.623; *P*=0.898 at *P*<0.05). Hence, it might be possible to combine high folate content with high biomass yield in a domestic variety/cultivar.

### Accumulation of folate precursor

**Fig. 5.**
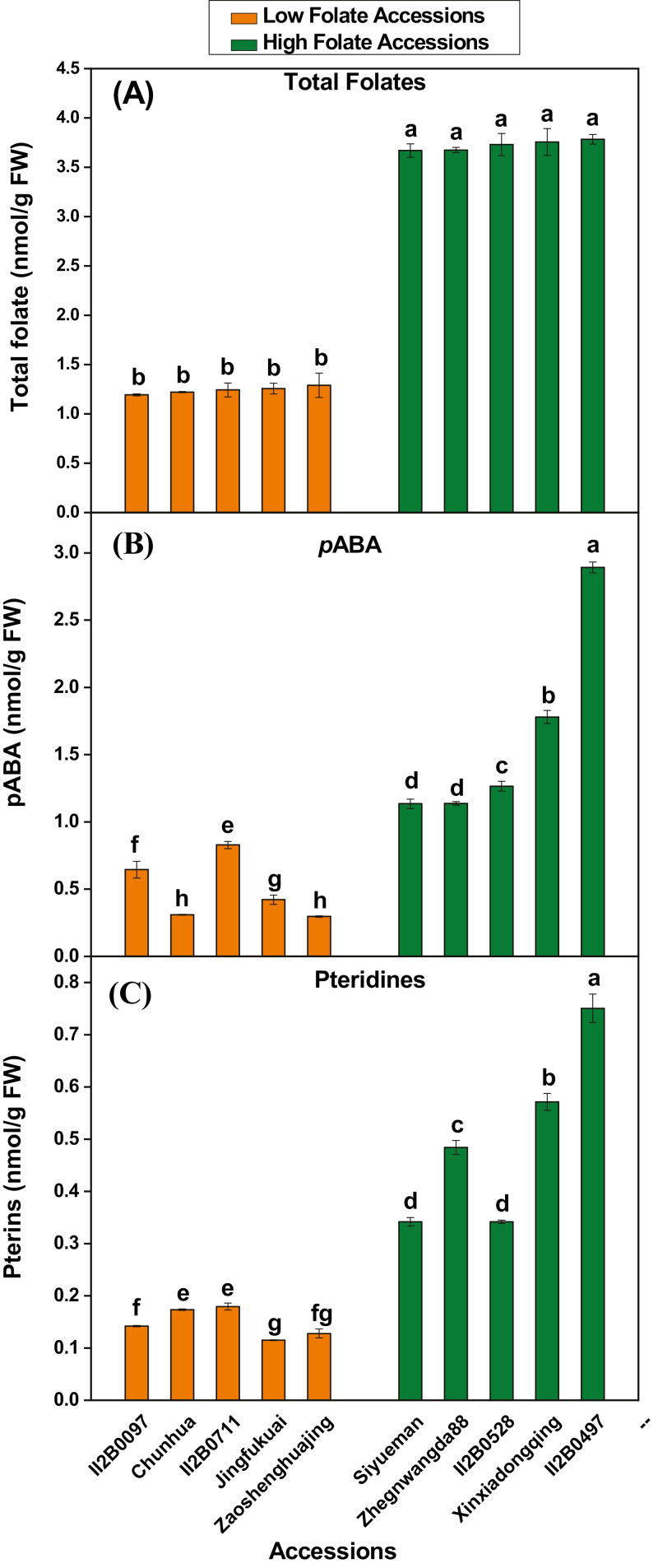
Folate precursor content measured in the high and low folate pak choi (*Brassica rapa* subsp. *Chinensis*) accessions/cultivars grown under controlled conditions. (A) Total folate, (B) *p*ABA, and (C) Pteridine content measured in high and low folate pak choi accessions/cultivars grown under controlled conditions. Each value is the average of three analyses from three independent replicates ±SD. Different letters at the top of bars within the same group indicate a significant difference at *P*<0.05 by Duncan’s multiple-range test.

Pteridine and *p*ABA are the main precursors for folate biosynthesis. Pteridine is synthesized in the cytosol, while *p*ABA is synthesized in plastids. These two moieties are condensed in mitochondria to form folate. To dissect the physiological basis of folate variation in diverse pak choi accessions, we have selected the highest five and lowest five folate content accessions (Fig. 5A) for further study. Levels of *p*ABA and pteridine accumulation were examined in the 10 selected accessions/cultivars which contain either high or low levels of folate. The range of total *p*ABA concentration in low folate and high folate accessions varied from 0.29 nmol gFW^–1^ to 0.82 nmol gFW^–1^, and from 1.13 nmol gFW^–1^ to 2.89 nmol gFW^–1^, respectively (Fig. 5B). On average, 3.28-fold variations in *p*ABA concentration were found in low folate and high folate accessions. Total pteridine concentration in low folate and high folate accessions ranged from 0.11 nmol gFW^–1^ to 0.18 nmol gFW^–1^, and from 0.34 nmol gFW^–1^ to 0.75 nmol gFW^–1^, respectively (Fig. 5C). On average, 3.37-fold variations in pteridine concentration were found in low folate and high folate accessions. A linear significant positive correlation between total folate and pteridine or *p*ABA was observed, with a correlation coefficient (*r*) of 0.77 (*P*=0.0095) and 0.85 (*P*=0.0016), respectively for *p*ABA and pteridine (Supplementary Table S8).

### Activity of enzymes related to folate metabolism

**Fig. 6.**
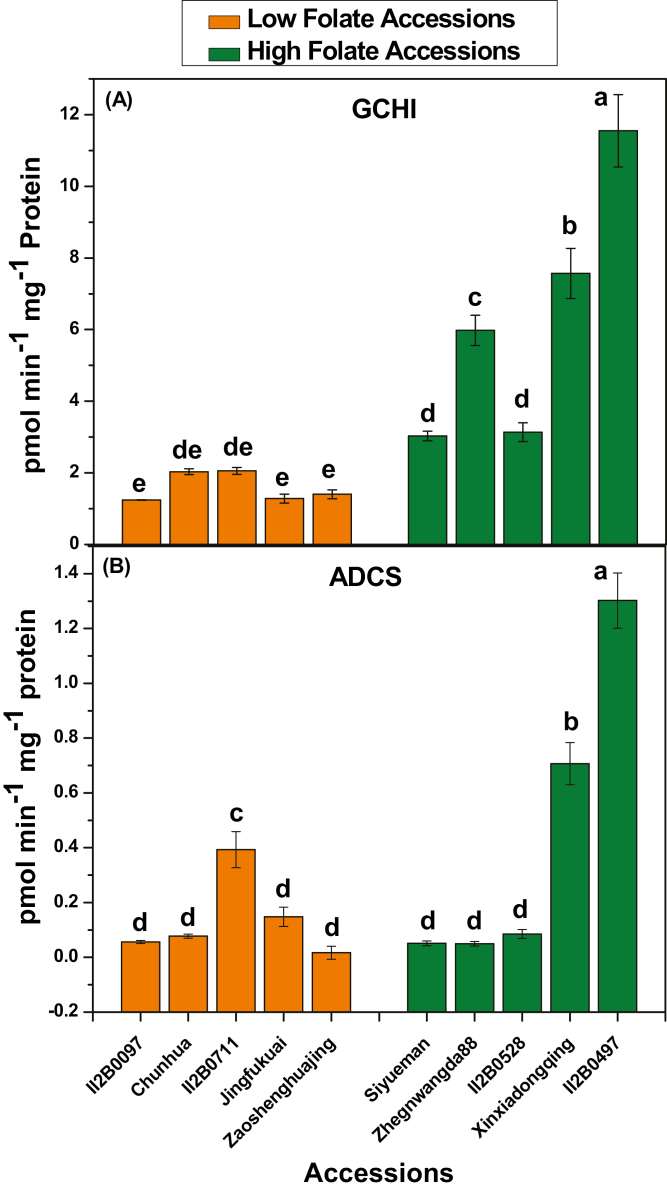
Assays of folate biosynthesis enzyme activities in high and low folate pak choi (*Brassica rapa* subsp. *Chinensis*) accessions/cultivars grown under controlled conditions. (A) GCHI activity assay. (B) ADCS activity assay. Each value is the average of three analyses from three independent replicates ±SD. Different letters at the top of bars within the same group indicate a significant difference at *P*<0.05 by Duncan’s multiple-range test.

The first step of pterin and *p*ABA biosynthesis is of particular interest because they control the flux in to the pathway. These steps were mediated by GCHI (EC 3.5.4.16) and ADCS (EC 2.6.1.85). The activity of pak choi GCHI and ADCS was determined. GCHI activity in the low folate and high folate accessions ranged from 1.24 pmol min^–1^ mg^–1^ protein to 2.05 pmol min^–1^ mg^–1^ protein and from 3.03 pmol min^–1^ mg^–1^ protein to 11.55 pmol min^–1^ mg^–1^ protein, respectively (Fig. 6A). One average, 3.9-fold variation in GCHI activity was found in low folate and high folate accessions. The range of ADCS activity in low folate and high folate accessions varied from 0.01 pmol min^–1^ mg^–1^ protein to 0.39 pmol min^–1^ mg^–1^ protein and from 0.05 pmol min^–1^ mg^–1^ protein to 1.3 pmol min^–1^ mg^–1^ protein, respectively (Fig. 6B). On average 3.17-fold variation in ADCS activity was found in low folate and high folate accessions.

### Expression of genes involved in folate metabolism

**Fig. 7.**
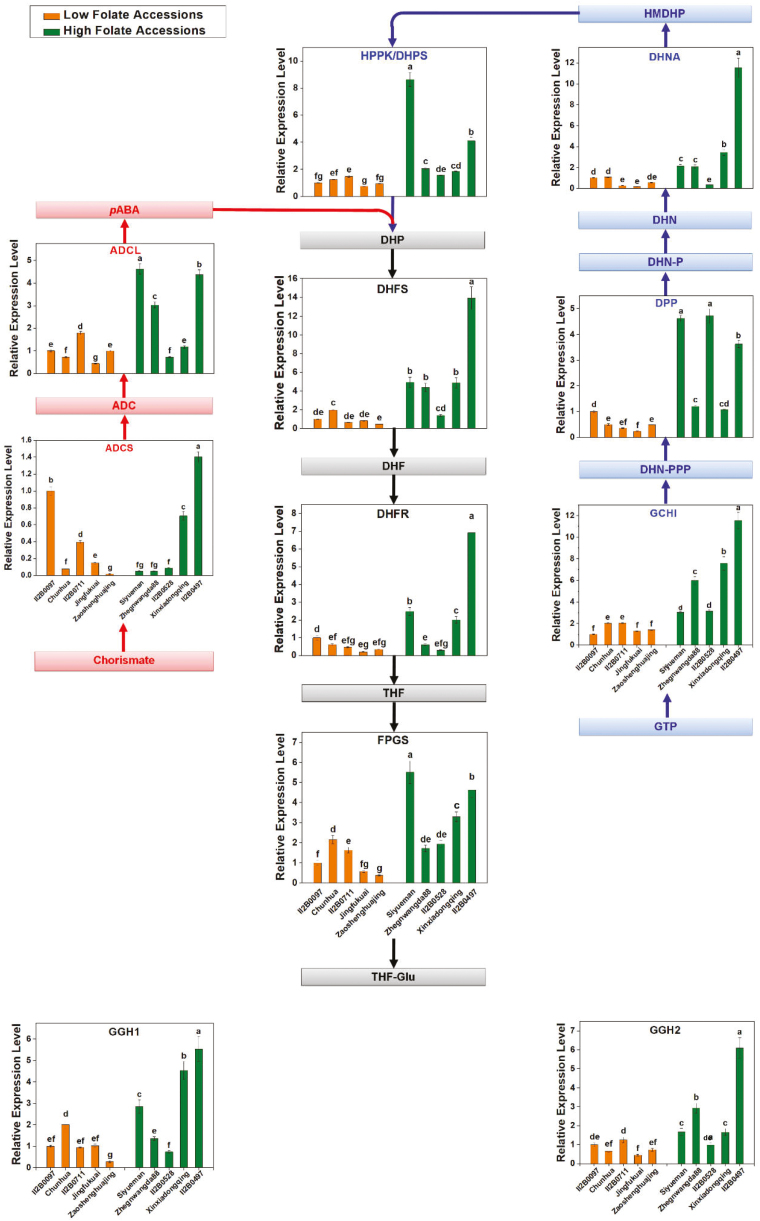
Relative expression of genes involved in folate metabolism in the high and low folate pak choi (*Brassica rapa* subsp. *Chinensis*) accessions/cultivars grown under controlled conditions. Metabolite abbreviations: ADC, aminodeoxychorismate; *p*-aminobenzoate, *p*ABA; DHNPPP, dihydroneopterin triphosphate; DHN-P, dihydroneopterin phosphate; DHN, dihydroneopterin; HMDHP, hydroxymethyldihydropterin; HMDHP-PP, hydroxymethyldihydropterin pyrophosphate; DHP, dihydropteroate; DHF, dihydrofolate; THF, tetrahydrofolate; THF-Glu, tetrahydrofolate polyglutamate. Gene abbreviations: *ADCS*, *ADC synthase*; *ADCL*, *ADC lyase*; *GCHI*, *GTP cyclohydrolase I*; *DPP*, *dihydroneopterin triphosphate pyrophosphatase*; *DHNA*, *dihydroneopterin aldolase*; *HPPK*-*DHPS*, *hydroxymethylpterin pyrophosphokinase-dihydropteroate synthase*; *DHFS*, *dihydrofolate synthase*; *DHFR*, *dihydrofolate reductase*; *FPGS*, *folylpolyglutamyl synthase*; *GGH1*, *gamma-glutamyl hydrolase 1*; *GGH2*, *gamma-glutamyl hydrolase 2.* Data are relative expression levels of mRNA determined by qRT-PCR using the ΔΔCt quantification method and an *actin* mRNA as internal reference. Each value is the average of three analyses from three independent replicates ±SD. Different letters at the top of bars within the same group indicate significant difference at *P*<0.05 by Duncan’s multiple-range test.

Prior to this work, pak choi cDNAs were unavailable for studying the enzymes of folate synthesis. Therefore, DNA sequences corresponding to these enzymes were identified by TBLASTN searches (https://blast.ncbi.nlm.nih.gov/Blast.cgi?PROGRAM=tblastn&PAGE_TYPE=BlastSearch&LINK_LOC=blasthome) of pak choi EST and genome databases using cognate Arabidopsis proteins as queries. This approach provided DNA sequences for a full set of folate synthesis genes, two of which (*DHNA* and *ADCL*) were present as two copies and *FPGS* was present as three copies. Only one copy of both *DHFS* and *DHFR* was found in pak choi, whereas three of each were present in Arabidopsis. Only the cytosolic, plastidial, and mitochondrial genes *DHNA*, *ADCL*, and *FPGS*, respectively, were chosen based on the resemblance of their products to the DHNA, ADCL, and FPGS isoforms in the corresponding Arabidopsis organelles (Table 1). The obtained DNA sequences were used to design primers for qRT-PCR (Supplementary Table S5). Folate pathway transcript variations were monitored in naturally selected high and low folate content pak choi accessions to investigate coordinate control of the folate synthesis genes. The relative transcription levels of the nine genes (*GCHI*, *DPP*, *DHNA*, *HPPK/DHPS*, *ADCS*, *ADCL*, *DHFS*, *DHFR*, and *FPGS*) associated with folate biosynthesis and two genes (*GGH1* and *GGH2*) associated with the hydrolysis of polyglutamylated folate or *p*ABA, a folate precursor coding for folate biosynthesis, were analyzed in 10 selected accessions which contain either high or low levels of folate as determined by qRT-PCR (Fig. 7). Overall, all the genes were expressed in all high and low folate content pak choi accessions. In general, folate biosynthesis genes were found to be expressed at high levels in high folate content accessions but at low levels in low folate content accessions. However, when individual accessions/cultivars were compared, our data provide a mixture of evidence for regulation of folate biosynthesis genes (Fig. 7). The present study confirmed the expression patterns, but showed that they are not mirrored by other folate synthesis genes. These data thus provide no proof for coordinate control of the whole set of folate pathway genes. The absolute levels of the various transcripts varied markedly, the lowest being *ADCS* and the highest *DHFS.* The high *DHFS* transcript abundance fits with the high activity of *DHFS* relative to other enzymes of folate biosynthesis, associated with the substantial flux involved in recycling the DHF produced during thymidylate synthesis. It should also be noted that, *DPP*, *ADCL*, and *FPGS* were very weakly expressed. A similar expression pattern was also observed in the case of the *GGH* genes; *GGH1* in particular was more highly expressed in high folate content pak choi accessions/cultivars.

**Table 1.** Folate metabolism enzymes and genes in Arabidopsis and *Brassica rapa*

Enzyme	Abbreviation	Arabidopsis gene^*a*^	*Brassica rapa* unigene^*a*^
GTP cyclohydrolase I	GCHI	AT3G07270	EX061901
Dihydroneopterin triphosphate pyrophosphohydrolase	DPP	AT1G68760	EX124881
Dihydroneopterin aldolase	DHNA	AT3G21730	EX123167
6-Hydroxymethyl-7,8-dihydropterin pyrophosphokinase/7,8-dihydropteroate synthase	HPPK/DHPS	AT4G30000	EX094965
4-Amino-4-deoxychorismate synthase	ADCS	AT2G28880	-
4-Amino-4-deoxychorismate lyase	ADCL	AT5G57850	ES934647
Dihydrofolate synthetase	DHFS	AT5G41480	EX039842
Dihydrofolate reductase/Thymidylate synthase	DHFR-TS	AT2G16370	EE525985
Folylpolyglutamate synthase	FPGS	AT3G10160	EX118503
Gamma-glutamyl hydrolase 1	GGH1	AT1G78660	EX121415
Gamma-glutamyl hydrolase 2	GGH2	AT1G78680	EE518174

^*a*^ Folate metabolism isoform.

### C1 metabolites and chlorophyll content

**Fig. 8.**
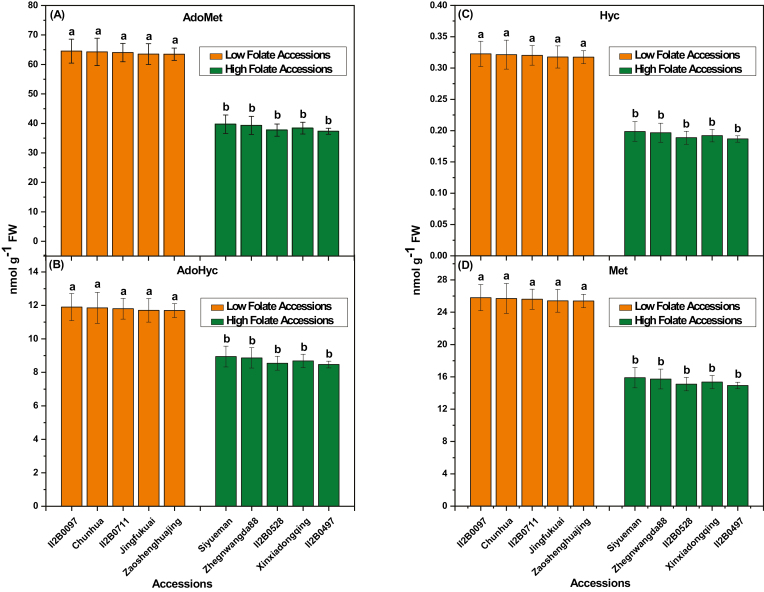
C1 metabolite content measured in the high and low folate pak choi (*Brassica rapa* subsp. *Chinensis*) accessions/cultivars grown under controlled conditions. (A) *S*-adenosylmethionine (AdoMet), (B) *S*-adenosylhomocysteine (AdoHcy), (C) Homocysteine (Hyc), and (D) Methionine (Met) concentrations were determined in the high and low folate pak choi accessions/cultivars grown under controlled conditions. Each value is the average of three analyses from three independent replicates ±SD. Different letters at the top of bars within the same group indicate a significant difference at *P*<0.05 by Duncan’s multiple-range test.

**Fig. 9.**
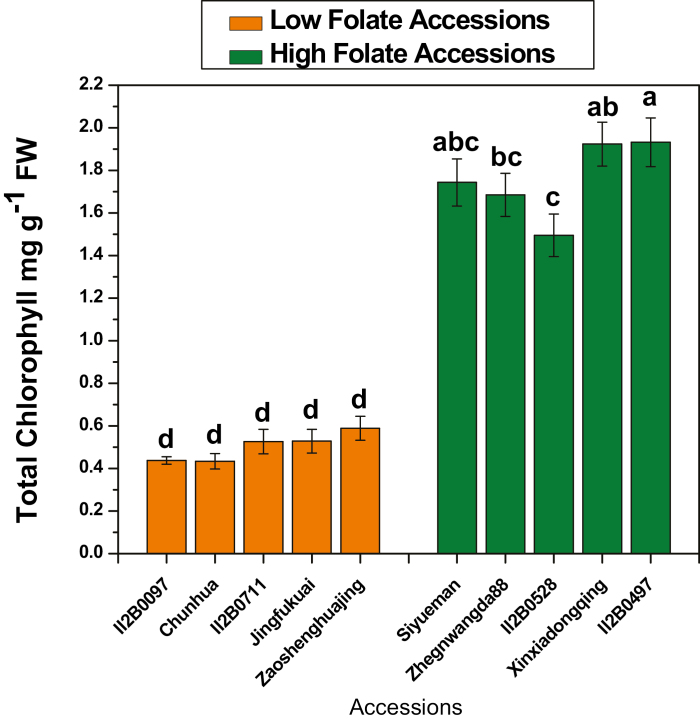
Chlorophyll content measured in the high and low folate pak choi (*Brassica rapa* subsp. *Chinensis*) cultivars/accessions grown under controlled conditions. Each value is the average of three analyses from three independent replicates ±SD. Different letters at the top of bars within the same group indicate a significant difference at *P*<0.05 by Duncan’s multiple-range test.

Derivatives of tetrahydrofolate molecules are primary cofactors of C1 metabolism. They are involved in various biosynthetic pathways, depending on the transported C1 unit. (Rebeille *et al.*, 2006). Since Met is the source of AdoMet, the universal methyl donor, folate derivatives are the basis of almost all C1 metabolism reactions (Hanson and Roje, 2001). The AdoMet concentration was high in low folate pak choi accessions and low in high folate pak choi accessions (Fig. 8A). On average 1.66-fold variations in the AdoMet concentration was found in low folate and high folate accessions. Similarly, AdoHcy concentration was high in low folate accessions and low in high folate accessions (Fig. 8B). On average 1.35-fold variation in AdoHyc concentration was found in low folate and high folate pak choi accessions. Nevertheless, the AdoMet/AdoHcy ratio, often referred to as the methylation index, was 6.5. Hcy and Met are the other two primary intermediates of the methyl cycle. The Hyc concentration was 1.66-fold higher in low than in high folate pak choi accessions (Fig. 8C). In contrast, the Met concentration was 2.38-fold higher in low folate than in high folate pak choi accessions (Fig. 8D). Contrasting results were observed in the case of total chlorophyll content (Fig. 9). Total chlorophyll content was high in high folate accessions and low in low folate accessions, ranging from 0.43 mg gFW^–1^ to 0.58 mg gFW^–1^ and from 1.5 mg gFW^–1^ to 1.93 mg gFW^–1^, respectively. On average 3.49-fold variation in total chlorophyll content was found in low folate and high folate pak choi accessions. A linear significant positive correlation (*r*=0.989; *P*=0.000000128) was observed between total folate and chlorophyll content (Supplementary Table S9).

## Discussion

Increasing the natural folate content in food crops is an efficient way to fight against worldwide prevalent folate deficiency, in contrast to the current intervention of pharmaceutical supplementation and flour fortification (Bhutta and Salam, 2012). Recent research shows that consumption of synthetic folic acid can affect the levels and patterns of DNA methylation in humans, accelerate the prevalence of certain types of cancers, and mask vitamin B_12_ deficiency (Cole *et al.*, 2007; Bae *et al.*, 2014; Moore *et al.*, 2014). In addition, populations with a homozygous mutation in the methylenetetrahydrofolate reductase gene (677TT genotype) cannot control their blood homocysteine level even when supplied with optimum synthetic folic acid (Moore *et al.*, 2014). Thus, it is more important to take in natural folates from edible parts of food crops.

Among the leafy green vegetables, pak choi is an important source of phytonutrients in the diet, comparable with spinach in its total folate content (Shohag *et al.*, 2012b; USDA, 2019). However, folate content in pak choi is not enough to supply the RDA (recommended daily allowance) from a single serving. The development of crop plants, such as pak choi varieties, to deliver a large proportion of the RDAs of folate (biofortification) is a prime goal for plant breeders. To date, little is known about the extent of folate content in diverse pak choi germplasms. To address this issue, 74 pak choi accessions/cultivars that were collected from diverse geographical origins from different horticultural and cultivar groups were grown under controlled-environmental conditions and screened for their natural diversity of folate content.

Total folate content of pak choi was investigated in previous studies, varying from 66–333 μg 100 gFW^–1^ in a microbiological assay (Iwatani *et al.*, 2003; USDA, 2019) to 90–145 μg 100 gFW^–1^ by the LC method; (Houlihan *et al.*, 2011; Shohag *et al.*, 2012b). However, no study investigated large numbers of accessions under control environmental conditions. In general, the folate content analyzed by LC was 20–52% lower than that for the microbiological assay (Ruggeri *et al.*, 1999; Konings *et al.*, 2001; Kariluoto *et al.*, 2006). Therefore, analysis of certified reference materials and confirmation of the method validation is necessary to reduce methodological dissimilarities. The analytical performance in the present study was evaluated by recovery test, precision, linearity, sensitivity, and analysis of certified reference materials. The recovery and precision ranged from 71.27% to 99. 01% and from 1.7% to 7.8% RSD, respectively; the limit of detection and limit of quantification ranged between 0.003 µg 100 g^–1^ and 0.021 µg 100 g^–1^ and between 0.011 µg 100 g^–1^ and 0.041 µg 100 g^–1^, respectively, depending on the different folate vitamers. Additionally, we examined the reference material BCR-485 for method validation. The certified value of total folate in BCR-485 by the microbiological assay was 315±28 µg 100 g^–1^, and the value of total folate from our study was 289.3±1.5 µg 100 g^–1^ (*n*=10). However, all of the previous reports were mainly focused only on the amount of folate in pak choi, and there are no previous studies with such a large number of accessions/cultivars as screened in our study. In addition, all of the accessions/cultivars were grown in similar environmental conditions, as required for authentic comparisons of natural genetic variation. Thus, our study resulted in precious new information on the extent of natural genetic variation in folate content of pak choi germplasm.

In terms of stability, 5-CH_3_-H_4_folate and 5-HCO-H_4_folate were known to be more stable folate vitamers (O’Broin *et al.*, 1975; DellaPenna, 2007), which were contributing 68% of the total folate pool in pak choi. Moreover, 5-CH_3_-H_4_folate is the first choice of enhancement, because it is the only folate vitamer found in the human bloodstream (Scott *et al.*, 2000). Some previous reports with different sample preparation and detection methods showed some difference in the folate vitamer distribution in pak choi (Houlihan *et al.*, 2011) and also mention that 5-CH_3_-H_4_folate was the main folate vitamer in this vegetable. In agreement with this and consistent with data from our previous report (Shohag *et al.*, 2012b), our result demonstrate that H_4_-folate, 5-CH_3_-H_4_folate, 5-HCO-H_4_folate, and 10-HCO-folic acid account for 22, 55, 13, and 10% of the total folate pool of different batches of pak choi, respectively.

A diverse range of pak choi accessions/cultivars were compared in this highly controlled experiment. Interactions of biomass yield (DW and FW basis) and total folate content were also compared. There was no or minimal correlation (*r*=0.058; *r*=0.015) between these two parameters, and this was statistically insignificant (*P*=0.623; *P*=0.898 at *P*<0.05). This suggests that folate content and biomass yield might be independent characters; these two characters might be able to be improved independently. Hence, it might be possible to combine high folate content with high biomass yield in a domestic variety. Ward *et al.* (2008) reported that folate content did not clearly correlate with either bran yield (*r*= –0.127; *P*=0.121) or 100 grain weight (*r*=0.137; *P*=0.095) when 175 wheat lines were compared. Similar trends were also found in leaf yield and folate content in diverse spinach accessions (Shohag *et al.*, 2011).

Pak choi is a widely consumed leafy green vegetable worldwide and encompasses wide genetic diversity. The main purpose of this investigation was to explore the natural genetic variation of folate content among the pak choi accessions for the biofortification program, and to determine whether folate-rich accessions could be identified as a source of pak choi genes for boosting the folate content by smart breeding. Discovering which are high and low folate pak choi accessions may prove useful in understanding the physiological and molecular basis of folate variation in pak choi. In this controlled study, we observed that the folate content varied considerably among the accessions evaluated. A 3.2-fold variation in the total folate content was observed between the highest and lowest accessions, and a 4.5-fold variation was found in the concentration of the predominant vitamer, 5-CH_3_-H_4_folate. Nevertheless, it might be possible that crossing a high folate line with the most stable and bioavailable vitamers could be effective, because no breeding efforts have previously been made toward specifically increasing folate in pak choi. Apart from a transgenic approach, several approaches, such as a quantitative trait locus (QTL) study, may also be possible, since there are many more existing accessions than those which we have investigated in this study, and natural genetic variation may be higher than what we observed.

This study was a first step towards folate biofortification in a widely consumed leafy green vegetable, pak choi. A folate concentration as high as 166.9 μg 100 gFW^–1^ was measured in pak choi accessions, which is close to that found in transgenic tomato (180.5 μg 100 g^–1^) (Díaz de la Garza *et al.*, 2004) and lettuce (188.5 μg 100 g^–1^) (Nunes *et al.*, 2009). Cooking loss reported in literature data demonstrate ~40% of pak choi folate loss after 30 s boiling (Han *et al.*, 2005). Thus a serving of the highest folate-rich pak choi accession alone can contribute ~50% of the dietary reference intake (DRI) for adults (400 μg d^–1^) and 35% of the DRI for pregnant women (600 μg d^–1^) without any modification (NDB_ No.11458: 190 g cooked, according to USDA, 2019).

The activities of GCHI and ADCS were high in high folate and low in low folate pak choi accessions, respectively. These results are consistent with the enzyme activity in wheat, where GCHI and ADCS activities were high in folate-rich tissue (McIntosh *et al.*, 2008). In general, the amount of pteridine and *p*ABA determines the amount of total folate in the plant. In the present study, a linear, significant positive correlation between total folate and pteridine or *p*ABA was observed, which is in agreement with previous reports (Díaz de la Garza *et al.*, 2007). However, when precursor levels (Fig. 5B, C) and enzyme activities (Fig. 6A, B) in the individual pak choi accessions/cultivars were compared, there was a little confusion regarding the regulation of biosynthesis of *p*ABA, pterin, and total folates (Fig. 5A), since the levels of these compounds stay within characteristic ranges (Jabrin *et al.*, 2003; Orsomando *et al.*, 2006; Hanson and Gregory, 2011) for different tissues, growing conditions, and genotypes, which implies that the regulatory mechanisms vary genetically. This scenario can be explained by the plausible biochemical feedback control of the upper and lower part of this pathway (Fig. 1B). Sahr *et al.* (2006) reported that the first enzyme of *p*ABA synthesis, ADCS, was inhibited by DHF and its precursor DHP. Mouillon *et al.* (2002) showed that the enzyme that couples pterin and *p*ABA, DHPS, was inhibited by the downstream pathway products. All folate metabolism genes were found to be expressed at high levels in the high folate content accessions but at low levels in the low folate content accessions. These results are in agreement with the expression pattern in potato, where mRNAs of folate biosynthesis genes were highly expressed in the folate-rich potato germplasm (Robinson *et al.*, 2019). There is so far only fragmentary evidence for folate-mediated regulation of folate synthesis genes in plants. Expression of the bifunctional *HPPK–DHPS* gene varies greatly during development in pea seedlings (Jabrin *et al.*, 2003), as did expression of *GCHI*, *ADCS*, *HPPK–DHPS*, and *FPGS* in wheat grains (McIntosh *et al.*, 2008) and expression of *GCHI*, *ADCS*, and *ADCL* in tomato fruit (Basset *et al.*, 2002). Moreover, *FPGS* and *GGH* play an essential role in folate homeostasis by polyglutamylation of folate. In Arabidopsis, it was found that ablation of the mitochondrial *FPGS* or overexpression of *GGH* in vacuoles caused 40–45% reduction in total folate, while lower total *GGH* activity increased total folate content by 34% (Akhtar *et al.*, 2010; Mehrshahi *et al.*, 2010). In a recent study, Robinson *et al.* (2019) showed that *GGH1* expression was consistently higher in high folate clones compared with low folate clones. Therefore, the expression pattern of *GGH* genes in our study suggests that total folate content is tightly regulated in individual pak choi accessions/cultivars. Moreover, expression levels of genes are not always reflected in their corresponding protein levels or enzyme activities. As additional regulatory mechanisms at the post-transcriptional level could also play an important role in the regulation of folate biosynthesis, further investigation of protein levels may be useful to determine which of the folate metabolism genes are responsible for variation in folate concentrations in the high and low folate germplasms.

5-CH_3_-H_4_folate is the main folate vitamer. This cofactor is essential for Met synthesis, because 80% of the Met pool participates in AdoMet synthesis and turnover (Giovanelli, 1987). The concentration of a metabolite depends on the equilibrium between its production and utilization rates. A higher concentration of Hcy strongly suggests that its conversion into Met is hampered, as expected in the case of 5-CH_3_-H_4_folate shortage. However, the fact that the Met concentration remained high is also indicative of either a lower Met utilization (through the methyl cycle and/or protein synthesis) or a higher *de novo* synthesis. Indeed, *de novo* synthesis of Met is located in plastids (Ravanel *et al.*, 2004) and is not, therefore, directly connected with the methyl cycle in the cytosol. From this point of view, it is possible that a ‘rescue’ system takes place in such a situation in order to maintain Met and AdoMet concentrations. Such a system was observed in Arabidopsis cells where severe folate deficiency occurred and, AdoMet and Met concentrations were rapidly restored after a temporary drop (Loizeau *et al.*, 2007). This metabolic control possibly involves complex regulatory mechanism of *de novo* Met synthesis in plastids, where cystathionine-γ-synthase plays a key role (Hacham *et al.*, 2002; Loizeau *et al.*, 2007).

In nature, chlorophyll biosynthesis is one of the major biosynthetic pathways. To synthesize the tetrapyrrole macrocycle ring of Chl *a*, just three precursors are needed: a methyl group, an Mg^2+^ ion, and eight molecules of glutamate. They are joined to a phytol chain and Chl *a* is synthesized via the isoprenoid pathway. Studies on the relationship between the isoprenoid and tetrapyrrole pathways revealed that the three precursors were closely linked (Laule *et al.*, 2003). In recent years, attention has been paid to the coordination of production of chlorophyll and chlorophyll-binding proteins (Tanaka and Tanaka, 2007). To date, however, there is minimal information available as to how the pathway integrates with C1 metabolism for the supply of methyl groups, required for the conversion of Mg-protoporphyrin IX to Mg-protoporphyrin IX methyl ester by Mg-protoporphyrin IX methyltransferase. The methyl group is derived from AdoMet, which in turn obtains its methyl group from the folate pool. In agreement with the correlation between folate and chlorophyll content revealed in our study, a close link between chlorophyll biosynthesis and folate was also demonstrated in a previous report by Van Wilder *et al.* (2009). It is likely in the future that a systems approach to studying plant folate metabolism will reveal many as yet unrecognized links between different pathways, shedding some light on their evolution and facilitating informed metabolic engineering.

### Potential nutritional impact of high folate pak choi germplasm

The nutrient composition of the five selected high folate folate pak choi germplasms were assessed (Table 2). High folate germplasms also contain substantial amounts of total carotenoid (1894 µg g^–1^ in II2B0497), vitamin C (264.98 mg kg^–1^ in II2B0497), total glucosinolates (22.18 µg g^–1^ in Xinxiadongqing), total protein (54.88 mg g^–1^ in II2B0497), calcium (26.91 mg g^–1^ in Siyueman), iron (129.81 mg kg^–1^ in Siyueman), zinc (72.79 mg kg^–1^ in II2B0497), and selenium (1.25 mg kg^–1^ in II2B0497). The concentrations of vitamins and minerals we observed compare favorably with their RDIs; a single serving of 150 g of cooked (one cup) pak choi would provide more than half of the RDI of all vitamins and minerals mentioned above.

**Table 2.** Nutrient composition of the five selected high folate pak choi (*Brassica rapa)* germplasms

Nutrients	High folate pak choi (*Brassica rapa)* germplasm				
	II2B0497	Xinxiadongqing	Ⅱ2B0528	Zhegnwangda88	Siyueman
Total carotenoid (µg g^–1^)	1894±38.68 a	1433±18.3 c	178±23.24 e	994±13.81 d	1594±13.81 b
Vitamin C (mg kg^–1^)	264.98±0.33 a	243.22±18.22 ab	211.13±37.91 b	219.19±25.69 b	133.79±11.12 c
Total glucosinolates (µg g^–1^)	18.36±0.32 c	22.18±1.11 a	18.68±0.85 c	13.96±0.22 d	20.34±0.98 b
Total protein (mg g^–1^)	54.88±0.43 a	50.3±1.05 b	42.18±1.85 c	37.75±1.21 d	48.68±1.08 b
Calcium (Ca) (mg g^–1^)	20.67±0.90 d	27.14±0.43 a	23.21±0.23 c	24.87±0.92 b	26.91±1.32 a
Iron (Fe) (mg kg^–1^)	90.47±13.74 bc	102.39±6.85 b	78.71±2.33 c	63.62±5.73 d	129.81±7.09 a
Zinc (Zn) (mg kg^–1^)	72.79±5.04 a	44.42±1.39 c	66.37±0.65 b	38.96±0.96 c	41.53±3.72 c
Selenium (Se) (mg kg^–1^)	1.25±0.01 a	1.04±0.02 c	1.18±0.01 b	1.17±0.01 b	1.18±0.01 b

The results are expressed as means ±SD. Different letters after means within the same row indicate a significant difference at *P*<0.05 by Duncan’s multiple-range test based on four independent determinations of each composite sample.

One matter that needs to be considered is the bioavailability of the observed micronutrients. In most micronutrient-enhanced plants reported to date, assumptions about whether the nutrients can be readily absorbed by humans have not been fully tested (Jeong and Guerinot, 2008), although there are examples in which absorption studies have been carried out with mice and humans to show the direct nutritional benefits of mineral-enhanced crops (Morris *et al.*, 2008). In assessing strategies to deal with micronutrient deficiency, the provision of a varied diet with fresh meat, fish, fruit and vegetables would be ideal. However, where this varied diet is impossible because of poverty and poor governance, super enhanced, nutritionally complete vegetables could provide a durable solution to improve the health and general well-being of impoverished people. The social and economic impacts of nutritionally enhanced vegetables must be addressed, such as the cost-effectiveness of adapting local varieties (Ramessar *et al.*, 2008). Even so, there is no doubt that the nutritional qualities of plants can be enhanced by selection, breeding, or genetic engineering. The adoption of nutritionally improved pak choi could help to improve the health and well-being of the world’s poorest people.

In conclusion, this study provides new information about natural genetic variation of folate content and its physiological regulation in pak choi germplasm. The large number of pak choi accessions from diverse geographical origins and controlled experimental conditions provide a unique opportunity to obtain reliable information on folate diversity in pak choi accessions. We found that the folate content varied greatly among the accessions evaluated. The highest and lowest folate content accessions were evenly distributed among their geographical origins. The folate content and biomass yield could be increased independently, and it might be possible to combine both traits together. The transcriptional regulation of folate metabolism gene expression might contribute to the different levels of folate accumulation in pak choi germplasm. Folate diversity and chlorophyll content were tightly regulated through the methyl cycle. Chlorophyll content might be one of the biomarkers for high folate germplasm selection. Our screening experiment provides the fundamental basis for breeding folate-rich pak choi varieties and unravels the physiological basis of folate homeostasis in diverse plant species.

## Supplementary data

Supplementary data are available at *JXB* online.

Table S1. Pak choi (*Brassica rapa* subsp. Chinensis) accessions/cultivars evaluated for folates in this study.

Table S2. Optimized 5 min gradient elution for UPLC–MS/MS analysis of four folate vitamers, folic acid, and internal standard.

Table S3. Selected multiple reaction monitoring (MRM) transitions and compound parameters for four folate vitamers, folic acid, and internal standard.

Table S4. Optimized conditions for the tandem quadrupole (QqQ) mass detector for analysis of four folate vitamers, folic acid, and internal standard.

Table S5. Synthetic oligonucleotides used in this study.

Table S6. Optimized 7 min gradient elution for UPLC–MS/MS analysis of C1 metabolites.

Table S7. Ranking of pak choi (*Brassica rapa* subsp. *Chinensis*) accessions/cultivars in relation to total folate content.

Table S8. Pearson correlations analysis between total folate and *p*ABA or pterins.

Table S9. Pearson correlation coefficient analysis between total folate and total chlorophyll content.

eraa218_suppl_Supplementary_Table_S1

eraa218_suppl_Supplementary_Tables_S2-S8
