# Supplementary material for: Genetic and physiological regulation of folate in pak choi (Brassica rapa subsp. Chinensis) germplasm
Source: J Exp Bot. 2020 Jul 8;71(16):4914–29. doi: 10.1093/jxb/eraa218 (PMC7410185; doi:10.1093/jxb/eraa218)
Supplement: eraa218_suppl_Supplementary_Tables_S2-S8 [file eraa218_suppl_supplementary_tables_s2-s8.pdf]

SUPPLEMENTARY TABLE S2.

Optimized five-minute gradient elution for UPLC–MS/MS analysis of four folates vitamers, folic acid and internal standard.

Time (min)	Organic phase (%)	Aqueous phase (%)
Initial (0.00)	10.0	90.0
1.00	10.0	90.0
1.50	50.0	50.0
2.00	90.0	10.0
3.00	90.0	10.0
3.50	10.0	90.0
5.00	10.0	90.0

SUPPLEMENTARY TABLE S3.

Selected multiple reaction monitoring (MRM) transitions and compound parameters for four folates vitamers, folic acid and internal standard.

Compound name	Precursor ion (*m/z*)	Product ion (*m/z*)	Retention time (t_r)	Dwell time (*S*)	Cone voltage (*V*)	Collision energy (*V*)
H_4folate	446.2	166.4	0.48	0.019	22	42
		299.3		0.019	22	13
5-CH_3–H_4folate	460.0	194.0	0.51	0.019	22	35
		313.0		0.019	22	20
5-HCO–H_4folate	474.3	327.1	0.68	0.019	32	22
		166.1		0.019	32	44
		299.1		0.019	32	32
		120.0		0.019	32	36
10-CHO–PteGlu	470.0	175.9	0.64	0.019	27	30
		275.9		0.019	27	22
		295.2		0.019	27	28
PteGlu	442.0	176.0	0.75	0.019	22	27
		295.0		0.020	22	22
MTX	455.3	308.1	1.77	0.020	12	20
		175.1		0.020	12	38
		134.0		0.020	12	30
		106.0		0.020	12	72

SUPPLEMENTARY TABLE S4.

Optimized conditions for tandem quadrupole (QqQ) mass detector for analysis of four folates vitamers, folic acid and internal standard.

Source parameters	Value
Capillary voltage	3.12 kV
Source offset	25 V
Source temperature	150 °C
Desolvation gas temperature	400 °C
Desolvation gas flow	1000 L hr^{-1}
Cone gas flow	150 L hr^{-1}
Nebuliser gas flow	7.0 bar
Collision gas flow	0.15 mL min^{-1}

SUPPLEMENTAL TABLE S5

Synthetic oligonucleotides used in this study.

Gene abbreviations: GCHI, GTP cyclohydrolase I; DPP, Dihydroneopterin triphosphate pyrophosphohydrolase; DHNA, dihydroneopterin aldolase; HPPK/DHPS, 6-hydroxymethyl-7,8-dihydropterin pyrophosphokinase/7,8-dihydropteroate synthase; ADCS, 4-amino-4-deoxychorismate synthase; ADCL, 4-amino-4-deoxychorismate lyase, DHFS, Dihydrofolate synthetase; DHFR, dihydrofolate reductase; FPGS, folylpolyglutamyl synthase; GGH1, Gamma-glutamyl hydrolase 1, GGH2; Gamma-glutamyl hydrolase 1 ; ACT, actin.

Gene-specific primers			
Gene	*Forward primer* *(5´-3´)*	*Reverse primer* *(3´-5´)*	PCR size (bp)
GCHI	GGGCATCTGGAAAAATCTCA	ACCTTGCAGGAGAAGCTTGA	217
DPP	TCCTGCTCTACAACGAAACA	CTCATCAATCGGCAACTCCA	129
DHNA	AAGAGAGGGAATTGGGTGGT	TTGGCTTTCCGAACTTCACT	112
HPPK/DHPS	CGTCTCCTTCCACCGTTACTA	CTGTTTCCGATGTTGCTTCC	189
ADCS	CAGGTGCCTGATGCTACTCA	CTTTATGCGCAAGACGTTCA	211
ADCL	TTTCATTCCCTGAGCCTATC	CGACCTGAAGAACATGGAGC	100
DHFS	CTTTAAGGAGCCAAACACGC	CATTGGTGGAGGCAAGGTTA	130
DHFR	TTCCTCCCAAGTCCTCCTCA	TTTCCCGTCTTTACCGATGC	161
FPGS	GGCGACCGCTCACCTACCTT	TCCCTTCGTTCCTGCGACAT	126
GGH1	ACTAGATAGTTTGCTGCGTGTT	ATACCCAGTGACTGCCTTCC	120
GGH2	CTGCGGCGATTCTCCTTCCT	ACCTACCCGTCGCTCCGTCT	129
Actin	TATTCAGCCACTCGTTTGCG	GGACTGTGCCTCGTCACCAA	169

SUPPLEMENTARY TABLE S6.

Optimized seven-minute gradient elution for UPLC–MS/MS analysis of C1 metabolites.

Time (min)	Organic phase (%)	Aqueous phase (%)
Initial (0.00)	0.01	99.9
0.0-1.00	0.01	99.9
0.01-3.00	18.0	82.0
3.00-4.00	90.0	10.0
4.00-5.00	90.0	10.0
5.00-6.00	10.0	90.0
6.00-7.00	0.01	99.9

SUPPLEMENTARY TABLE S7.

Ranking of pak choi (*Brassica rapa* subsp. *Chinensis*) accessions/cultivars in relation to total folate content.

Group	Folate content (µg/100g fresh weight)	No. of Accessions	Name of Accessions
1	<55	3	II2B0097, Chunhua, II2B0711
2	55-69	6	Jingfukuai, Zaoshenghuajing, II2B0107, II2B0135, II2A0023, II2A0751
3	70-84	12	II2B1149, II2B0530, II2B1315, II2B1309, II2B0993, II2B0291, Meixia2, II2B0054, Hauxiahong, II2B1261, Beijingbaicai II2A0533
4	85-99	19	liuXing 8, Siji, Xiaozao 1, Beijingxin1, II2B0118, Youdonger, II2B0458, II2B0002, Lvbaoqinggeng, II2B0119, II2B0703, Shanghaiqing, Wuyueman, II2B0008, Qinggeng 60, Qingjiang328, II2B1251, II2B0198, Dianmei1
5	100-114	10	II2B0445, II2B0519, II2B0251, II2B0283, Aijiaonaibaicai, II2B0490, Meidu7030, II2B0267, Huaguan, Zhongjiaoheiyebai
6	115-129	9	Siji, Suzhouqing, II2B0574, II2B0668, Huoqing91-5c, Hualv 4, Wuyueman, II2A0752, II2B0310
7	130-144	5	Huangguanqingjiang, Baiyu1, II2B0599, II2B0059, Yusijixiaobaicai
8	145-159	4	Changgengbaicai, II2B0245, II2B030, Yusijixiaobaicai316
9	>160	6	Te’aiqingcai, Siyueman, Zhegnwangda88, II2B0528, Xinxiadongqing, II2B0497

In each group the accessions are given in order of increasing folate content.

SUPPLEMENTARY TABLE S8

Pearson correlations analysis between total folate and pABA or pterins.

		**Total Folate** (nmol/g FW)	***p*ABA** (nmol/g FW)	**Pterins** (nmol/g FW)
**Total Folate** (nmol/g FW)	Pearson Correlation	1	0.76768*	0.85542*
	p-value	-	0.00952	0.0016
***p*ABA** (nmol/g FW)	Pearson Correlation	0.76768*	1	0.94822*
	p-value	0.00952	-	0.0000295
**Pterins** (nmol/g FW)	Pearson Correlation	0.85542*	0.94822*	1
	p-value	0.0016	0.0000295	-

*: Correlation is significant at the 0.05 level

SUPPLEMENTARY TABLE S8

Pearson correlations coefficients analysis between total folate and total chlorophyll content.

		**Total Folate** (nmol/g FW)	**Total chlorophyll content** (mg/g FW)
**Total Folate** (nmol/g FW)	Pearson Correlation	1	0.98685*
	p-value	-	0.0000001287
**Total chlorophyll content** (mg/g FW)	Pearson Correlation	0.98685*	1
	p-value	0.0000001287	-

*: Correlation is significant at the 0.05 level
